# Trends and Patterns of Disparities in Oral Cavity and Pharyngeal Cancer in Serbia: Prevalence and Economic Consequences in a Transitional Country

**DOI:** 10.3389/fphar.2017.00385

**Published:** 2017-06-16

**Authors:** Gordana Djordjevic, Aleksandar Dagovic, Vladimir Ristic, Tatjana Kanjevac, Denis Brajkovic, Milica Popovic

**Affiliations:** ^1^Department of Epidemiology, University of KragujevacKragujevac, Serbia; ^2^Department of Clinical Oncology, Faculty of Medical Sciences, University of KragujevacKragujevac, Serbia; ^3^Department of Orthodontics, Faculty of Dentistry, University of BelgradeBeograd, Serbia; ^4^Department of Dentistry, Faculty of Medical Sciences, University of KragujevacKragujevac, Serbia

**Keywords:** oral carcinoma, prevalence, Europe, risk factors, National Health Programs

## Epidemiology of oral cavity and pharyngeal cancers

Oral cavity and pharyngeal cancers (OCPc) encompass malignancies arising in variety of anatomical subsites and are homogeneous regarding epidemiology, clinical presentation, associated risk factors and treatment modalities (Robinson and Macfarlane, 2003; Conway et al., 2006). Worldwide, they are the sixth most common type of cancer, with an estimated 529,500 new cases and 292,300 deaths globally during 2012, accounting for 3,8% of all cancer cases and 3,6% of cancer deaths (Ferlay et al., 2015; Shield et al., 2017). The regions with the highest incidence of OCPc include South and Southeast Asia (India, Pakistan, Sri Lanka), Eastern and Western Europe (Hungary, Slovakia, France, Portugal), Latin America (Brazil, Uruguay, Puerto Riko) and the Pacific (Papua New Guinea) (Warnakulasuriya, 2009; Chaturvedi et al., 2013; Weatherspoon et al., 2015). OCPc are the eighth most common cancers among the European countries with an incidence of 73,856 new cases and eleventh leading cause of cancer-related mortality with 34,285 death cases in the year 2012 (Ferlay et al., 2013). In the United States (US), an incidence of 41,380 newly diagnosed patients and 7,890 deaths due to OCPc was reported in 2013 (Weatherspoon et al., 2015). The incidence of OCPc has declined in recent decades in most parts of the world, but has increased in the US, United Kingdom, Australia, Canada, Sweden, Denmark and the Netherlands (Chaturvedi et al., 2013). OCPc are about three times more common in men than women, but this ratio is declining mainly due to increased exposure of women to risk factors (Warnakulasuriya et al., 2015). Recent study showed an increase of the incidence of OCPc for Serbian population for both men and women between 1999 and 2010 (Videnović et al., 2016). Age standardized incidence rates for OCPc for the Serbian population in 2012 were 11,2/100,000, which is lower compared to some European countries (Ferlay et al., 2013). The prevalence for men was 3,5 times higher than for woman, similar to trends in other parts of the Europe (Jankovic et al., 2006; Santric-Milicevic et al., 2009; Ferlay et al., 2013).

## OCPc risk factors

The traditional risk factors for OCPc are considered tobacco smoking and alcohol consumption, but strong evidences support the infection with high risk human papillomavirus (HPV) types 16 and 18, as an important causation factor (Marur et al., 2010; Chaturvedi et al., 2011; Radoï and Luce, 2013). Tobacco smoking and alcohol consumption are related to 75% of OPCc (Shield et al., 2017) and believed to have a synergistic effect (Cunningham et al., 2011). Reduction in smoking worldwide has resulted in significant declines in the incidence of oral cavity cancers in anatomical subsites such as lip, gum, floor of the mouth, hard palate, buccal mucosa, and vestibule (Tota et al., 2017). Evaluation by the International Agency for Research in Cancer (IARC) concluded the carcinogenicity of HPV type 16 in the oral cavity, oropharynx (including tonsil cancer, base of tongue cancer and other oropharyngeal cancer sites), and limited evidence for laryngeal cancer (IACR, 2012). HPV infection is accounted for more than 30% of OCPc in Northern and Western Europe (De Martel et al., 2012) and more than 60% in the United States (De Souza et al., 2011), compared to less than 10% in economically undeveloped countries (Chaturvedi et al., 2013). The incidence of HPV-infected OCPc is as much as 64% in Serbia (Kozomara et al., 2005). Currently immunization against the most common HPV types is not obligatory in Serbia, although the immunization against HPV type 16 of both men and women can prevent a large proportion of oropharyngeal cancers (IACR, 2012).

Much has been done in the Serbian legislature after 1999 in regards to preventive anti-smoking activities since Serbia had a reputation of having a very high smoking prevalence (Djikanovic et al., 2011). The Serbian health ministry ratified the World Health Organization (WHO) Framework Convention on Tobacco Control (Ministry of Health, 2007). The Law on Protection of Population from Exposure to Tobacco Smoke was passed in the Republic of Serbia in 2010, determining the measures of restricted use of tobacco products (Official Gazette of RS, 2010). However, the results of the National Health Survey in 2013 revealed that the smoking rate had increased by 3% after the year 2006 (Ministry of Health, 2014). In 2013 34,7% of the population were smokers with higher prevalence for men, but significant increase in the number of daily women smokers had been observed (Ministry of health 2014). Alcohol drinking in Serbia represents socialy acceptable behavior and alcohol use is a part of tradition and culture (Jakovljevic et al., 2013). According to WHO statistics the drinking pattern in Serbia has been scored 3 on a scale of 1–5, where 5 represents the riskiest drinking pattern (Jakovljevic et al., 2013).

## The economic burden of cancer treatment on Serbia's health system

The Republic of Serbia is a middle-income European country with high unemployment rates and decrease in growth of gross domestic product (GDP) (Santric-Milicevic et al., 2016). The health system in Serbia was based off of the health system of former SFR Yugoslavia, referred to as the Stampar model (Bredenkamp et al., 2011). This system has changed substantially after Yugoslavia's dissolution in the 90's, especially after democratic changes in the 2000 (Jakovljevic and Getzen, 2016; Jakovljevic et al., 2016; Mihailovic et al., 2017). Between 2006 and 2013 health care expenses rose from 9 to 10.4% GDP (The World Bank, 2016). More than half of public health expenditure is spent on salaries while only 3% is spent on capital investments and 6% on preventive health services (Santric-Milicevic et al., 2016).

Cancer reporting is obligatory by law in Serbia since 1986 (Miljuš et al., 2010). The Population Register for Cancer was formed in Serbia in 1970, which originated from the Plan of Statistical Research of Interest for the Republic (Official Gazette of the Republic of Serbia No. 32/69). Cancer reports were collected separately by the two cancer registries of Serbia: the Cancer Registry of Central Serbia and the Cancer Registry of Vojvodina (Mihajlović et al., 2013). Until the year 1998, the quality of data collection from these registries was insufficient. But in 1998 they both became members of IACR and the European Network of Cancer Registries which significantly improved the quality of the data (Miljuš et al., 2010).

In the US the 2008 health expenditures in treatment of malignancies reached 77,4 billion dollars (American Cancer Society, 2013) while EU costs for 2009 were 57 billion euros (Luengo-Fernandez et al., 2013). With the worldwide increase in cancer incidence, as well as developments in diagnostic and treatment procedures, these costs will constantly grow (Radovanovic et al., 2011). The burden of malignant diseases in Serbia is enormous (Jakovljevic et al., 2015). In a nation-wide population study on Serbian cancer incidence during 1999–2009 an alarmingly high incidence and mortality compared to European regions was reported (Mihajlović et al., 2013). A rising cancer incidence may be attributed to military conflict in the region, bombardment with depleted uranium during NATO campaigns, post war syndrome and poor socioeconomic parameters (Mihajlović et al., 2013; Kovacević et al., 2015). According to the last edition of the Statistical Yearbook of the Republic of Serbia, 21,865 people died of malignancies in the 2015 (Statistical Office of the Republic of Serbia, 2016).

In Serbia the diagnosis and treatment costs of cancer are dominated by radiotherapy related direct medical costs (Jakovljevic et al., 2015). Direct medical cancer care costs increased by 30% between 2007 and 2010 with radiotherapy consuming about 54% of all expenditures (Radovanovic et al., 2011). Regarding palliative cancer treatment the highest expenditures were observed for pharmaceutics (42%) (Kovacević et al., 2015). Economic recession and domestic policy constraints affected the affordability and quality of cancer treatment for the Serbian population (Dagovic et al., 2015). Lack of national screening programs, insufficient equipment capacities, poor network facilities across rural regions, late diagnosis and palliative care are the main issues to be solved in order to achieve better clinical outcomes and cost-effectiveness of the health care system (Jakovljević, 2013; Dagovic et al., 2015; Jakovljevic et al., 2015, 2017). Due to the economic recession, Serbia faces a migration of medical professionals (specialists, doctors, nurses), which may have potentially disastrous effects on quality of public health system (Santric-Milicevic et al., 2014, 2015, 2016).

## Economics of OCPc treatment

Early diagnosis of OCPc is vital to patient survival (Shield et al., 2017). Only one study assessed the effects of oral cavity cancer screening of high-risk groups, and reported that visual oral examination could reduce mortality by 29% (Sankaranarayanan et al., 2005). In many cases OCPc are diagnosed at advanced stages, when management is complex and multidisciplinary, and requires prolonged medical care (Hallenbeak et al., 2015). In the North American and European clinical practice, patients with early stage OCPc receive surgical treatment, patients with localy invasive disease are treated with combined treatment modalities (surgery, if indicated + radiotherapy and chemotherapy) while patients with end-stage metastatic disease usually receive palliative chemotherapy (Gold et al., 2009; Chan et al., 2012). Recontructive surgery, medical prostheses and multidisciplinary rehabilitation, follow up care and surveillance are required (Chan et al., 2012; Wissinger et al., 2014).

The full societal burden of cancer treatment consists of associated direct medical expenditures and indirect costs including reduced work force participation, loss of productivity and premature mortality (Wissinger et al., 2014). The existing data on costs associated with OCPc are available from a limited number of socioeconomic studies, mostly from the US and several from Europe. Summarized data from these studies suggest different pattern of expenses since costs associated with OCPc in the US were greatest for systemic therapy, while in the Europe the highest burden of costs was on surgical treatment (Preuss et al., 2007; Diaz-de-Cerio et al., 2013; Wissinger et al., 2014). In 2010 the direct medical costs of head and neck cancer treatment in the US totaled 3.64 billion dollars, which was slightly higher than indirect costs totaled 3.4 billion dollars (Mariotto et al., 2013). The mean per-patient direct medical costs for treatment of head and neck carcinomas in the US totaled 32,500–48,847 dollars, depending on the stage of disease (Wissinger et al., 2014). A report from Greece found that mean direct costs of treatment of OCPc were 7,450 dollars (Zavras et al., 2002). A similar study in the Netherlands reported direct per-patient costs of OCPc treatment of 22,080 dollars (van Agthoven et al., 2001). The main cost burden in patients with advanced disease is the need for multimodal treatments as well as the need for prolonged hospitalization and medical care (Wissinger et al., 2014). The costs of hospitalization of patients with OCPc in the US ranged approximately 20,000–23,000 dollars in 2012 (Le et al., 2013). Regarding the length of hospitalization, the average length of stay in US hospitals for OCPc patients was 16 days (Ryan and Hochman, 2000), considerably lower than in France (29 days) (Pinsolle et al., 1992), The Netherlands (31 days) (van Agthoven et al., 2001) and Greece (34 days) (Zavras et al., 2002). Regarding surgical treatment and defect reconstruction of OCPc, several studies from Europe, the US and Canada compared total costs within the first year of treatment after oral cavity reconstruction with microvascular flaps and local flaps, and observed minimal cost differences (Smeele et al., 2006; Le et al., 2013; Santamarta et al., 2013).

## Future implications to OCPc prevention

Although Serbian public health care expenditure is limited due to the recession, slow market recovery since 2013 has resulted with the substantial increase in oncology related public expenditure (Dagovic et al., 2015; Jakovljevic et al., 2015). Currently there are no data on the costs of treatment of OCPc in the Republic of Serbia and there is the need for further research on the budgetary costs. The future national strategies to reduce the prevalence of OCPc should include population health education targeted to reduce exposure to main risk factors: tobacco smoking, alcohol consumption and HPV infection. Promotion and finance of national screening programs, supported by legislure. may be one of the options to reduce cancer treatment costs and obtain more affordable health care.

## Author contributions

All six authors designed the research questions and concept of this opinion article. TK and DB acquired selected published data from the public registry European Health for All Data base issued by WHO. All six authors jointly interpreted the findings stated in the article and contributed important intellectual content to the final manuscript. All six authors checked English spelling and grammar.

### Conflict of interest statement

The authors declare that the research was conducted in the absence of any commercial or financial relationships that could be construed as a potential conflict of interest. The reviewer OZ declared a shared affiliation, though no other collaboration, with one of the authors VR to the handling Editor, who ensured that the process nevertheless met the standards of a fair and objective review.
